# Notes and News

**Published:** 1893-07-29

**Authors:** 


### Notes and News.

Collected and Communicated.

Field Marshal Sir Lintorn Simmons inspected the Gordon Boys at their Home last week. He congratulated them on their excellent health, not a single boy being in the hospital, and also on the conduct report. It is generally felt that the Home is fulfilling its object. In spite of the surroundings from which the boys are taken, an excellent report of them is given, both in the Home and after they leave it.

The Committee of the Central London Ophthalmic Hospital are making special efforts to obtain increased support for the institution. This is the Jubilee year of the hospital, and it is intended to commemorate the occasion by endeavouring to raise £30,000 to rebuild and enlarge the present premises. The Secretary will gladly receive contributions.

An important quarterly court was held recently at the Addenbrooke's Hospital, Cambridge. The state of the works was reported. The drainage was completed at the beginning of June, and a large portion of the hospital reopened by then. At this meeting new rules as to the admission of visitors were passed, and medical officers were re-elected, and new life governors were added to the list.

The British Home for Incurables may be well satisfied by the interest that has been evinced in its progress during the past year. It is true that annual subscriptions have shown a small decrease, but in other respects help has been most generous. The annual dinner and the garden party both held on its behalf added largely to its funds. "Two Sisters" founded the "Rest for the Weary" bed, giving £1,000 for the purpose. "A Lady" also presented the same sum to provide the "Penelope" bed. Altogether £5,297 was added to the general funds, and £4,542 to the building fund. The new home, of which the foundation-stone was laid by the Princess Christian a little over a year ago, will, it is expected, be shortly ready for occupation. The Princess of Wales, Patroness of the charity, has shown the greatest practical sympathy with its objects.

It is estimated that the Hospital Saturday Fund collection will not be equal to that of last year.

In these advertising days the proprietors of Mellin's Food for Infants must congratulate themselves on the mark of favour they have received, whereby their speciality has received the approbation of the Empress of Germany. Much pleased with its use in the Imperial Nursery, the Empress has issued a certificate to that effect under her hand and seal. Even in these democratic days not a few are found happy to follow in the footsteps of Royalty.

It is stated that the form of cholera now prevalent in Moscow is not of so fatal a nature as that of last year. The outbreak dates earlier than that of 1892. St. Petersburg is said to be now infected, and in Hungary and Roumania there are cholera cases also. Roumania has not yet joined the Dresden Sanitary Federation, as its acceptation of its conditions are dependent on the action of Turkey also in the matter. In England our sanitary authorities are showing commendable vigilance.

The Countess of Chichester opened the New Police Convalescent Home on the 21st inst., which has been erected in Bertram Road, Hove, at a cost of over £9,000, and the foundation-stone of which was laid a year ago by Princess Christian. Mr. Herbert Tritton presided, and amongst those present were Colonel Howard Vincent, M.P., the Rev. Prebendary Peacey, Vicar of Hove, and the Hon. the Rev. E. Carr Glyn, Vicar of Kensington. Over £7,000 has been subscribed towards the cost of the building, and in addition to the payments of the inmates about £550 will be required yearly to keep the home going. Any member of the police force of Great Britain and Ireland is eligible for admission. The Home will hold 52 beds, but only 32 of these are as yet supplied owing to lack of funds. Twenty-five convalescent policemen are at present in the Home.

At a meeting of the Royal Statistical Society held recently Dr. G. B. Longstaff read an interesting paper on "Rural Depopulation." The main object of the author was to give a broad view of the facts, to show in what countries rural depopulation existed and its degree. The case of the United Kingdom was examined in considerable detail. It was shown that the phenomenon appeared in 1851 in Wales, and speaking generally ten years later in England, the decrease in Scotland being considerably more noticeable. In Ireland it had also been severe since the time of the famine. The French and Irish birth-rates were equally low, though the marriage-rates differed greatly owing to the peculiar age distribution prevalent in Ireland as a result of emigration. Spain, Italy, Switzerland, and Norway all exhibited the phenomenon. The author claimed to have proved that not only was the disproportionate growth of large towns a phenomenon of universal prevalence, but that an actual diminution of the population occurred in the greater part of Europe, in North America, and even in Australia. The author held that nowadays there was a general and increasing aversion to country life, and that the general result could not be, in the main, at all events due to causes of only local occurrence, such as government, land laws, and the like.

